# Human chemosignals of disgust facilitate food judgment

**DOI:** 10.1038/s41598-018-35132-w

**Published:** 2018-11-19

**Authors:** Yan Zheng, Yuqi You, Ana R. Farias, Jessica Simon, Gün R. Semin, Monique A. Smeets, Wen Li

**Affiliations:** 1 Department of Psychology, Florida State University, Tallahassee, FL USA; 2 Center for Economics and Finance, Faculty of Economics, Universidade do Porto, Porto, Portugal; 3 Católica Research Centre for Psychological, Family and Social Wellbeing, Universidade Católica Portuguesa, Lisboa, Portugal; 4 William James Center for Research, ISPA Instituto Universitário, Lisboa, Portugal; 5 Department of Psychology, Utrecht University, Utrecht, The Netherlands

## Abstract

Choosing food is not a trivial decision that people need to make daily, which is often subject to social influences. Here, we studied a human homolog of social transmission of food preference (STFP) as observed in rodents and other animals via chemosignals of body secretions. Human social chemosignals (sweat) produced during a disgust or neutral state among a group of donors were presented to participants undergoing a 2-alternative-forced-choice food healthiness judgment task during functional magnetic resonance imaging (fMRI). Response speed and two key signal detection indices—*d’* (discrimination sensitivity) and *β* (response bias)—converged to indicate that social chemosignals of disgust facilitated food healthiness decisions, in contrast to primary disgust elicitors (disgust odors) that impaired the judgment. fMRI analyses (disgust vs. neutral sweat) revealed that the fusiform face area (FFA), amygdala, and orbitofrontal cortex (OFC) were engaged in processing social chemosignals of disgust during food judgment. Importantly, a double contrast of social signaling across modalities (olfactory vs. visual—facial expressions) indicated that the FFA and OFC exhibited preferential response to social chemosignals of disgust. Together, our findings provide initial evidence for human STFP, where social chemosignals are incorporated into food decisions by engaging social and emotional areas of the brain.

## Introduction

Every day, people make decisions about what to eat and what not to eat, exercising a keen effort on determining whether specific foods are healthy or not^1,2^. Through social media or personal conversations, food information is frequently offered and actively sought. In fact, social communication of food choices runs across the phylogeny. Non-primate animals use chemical secretions to communicate edibility and food choices among conspecifics^3–6^. In a well-established phenomenon of social transmission of food preference (STFP), mice would prefer a food consumed by other mice but only after smelling olfactory cues (e.g., carbon disulfide/CS_2_ on the breath of the other mice)^7,8^. The olfactory system is intrinsically associated with feeding^9^ and so it makes good sense that olfaction serves as an effective medium for food-related communication^8,10^.

Often deemed as a minor sensory system in comparison to other species, human olfaction nonetheless is documented behaviorally and neurally to possess extraordinary capacity for odor analysis^11–13^. For example, humans can potentially discriminate more than a trillion odors, way beyond their ability to discriminate colors (2.3–7.5 million) and tones (~340,000^11^). Furthermore, while visual cues seem to dominate human interactions, it is also true that a certain degree of modality-selectivity exists in social communication such that some sensory channels are better suited for transmitting some messages than others^14–17^). For example, touch predominantly communicates intimacy and complex emotions such as gratitude and sympathy while faces outperform in conveying basic emotions. We hypothesized that owing to its inherent association with feeding, the olfactory sense would be a privileged channel for food-related communication via human social chemosignals.

Human body odor, extracted largely from sweat, is a primary form of human social chemosignal, which has been shown to carry a wide range of information (e.g.,^18–21^). The recently flourishing research field on the communicative function of chemosignals has revealed that after smelling another person’s sweat produced during various behavioral and emotional states (e.g., anxious, fearful, or disgust), the receiver would display a simulacrum of the states and exhibit changes in cognition, affect, and behavior accordingly (e.g.,^20,22–29^). Among these emotions, disgust is a unique, ancient response to food, which is rooted in olfaction (and gustation), prompting an individual to avoid spoiled or poisonous food^30,31^. Therefore, sweat secreted in a disgust state could be a particularly useful social chemosignal for food and diet screening across people.

Concerning the neural mechanisms underlying social communication, much of our knowledge has come from research in the visual modality. Visual social transmission via facial expressions is known to involve limbic/paralimbic structures, including the amygdala, the anterior cingulate cortex (ACC), and ventral medial prefrontal cortex/orbitofrontal cortex (vmPFC/OFC), and the face-perception network (e.g., the fusiform face area/FFA; cf.^32,33^). Akin to their relevance to emotion and social communication, these regions are also nodes shared by the emotion network^34^ and the social network^35,36^. Although neural evidence concerning olfactory signaling of emotion remains relatively scant, converging evidence has implicated similar key structures of the social and emotion networks, including limbic/prelimbic areas and the FFA^26,29,37–40^. While these structures represent a core system, supporting amodal, abstract processing of social emotion, the neural system that collectively underpins olfactory communication (chemosignaling) of food choices has not been clearly defined.

**Figure 1:**
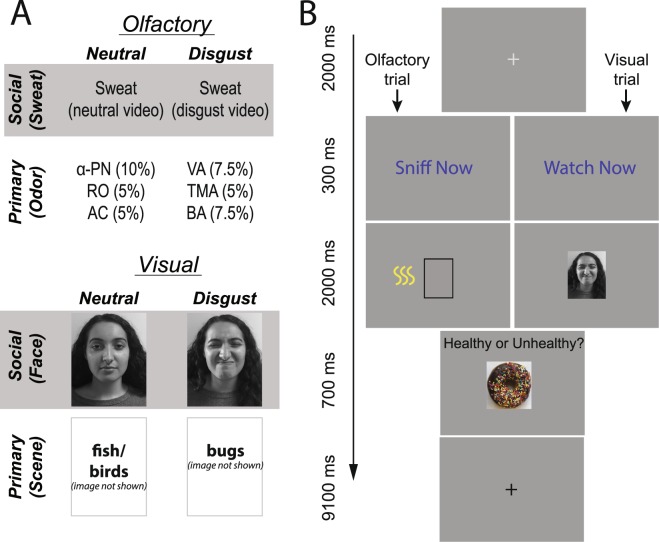
Experimental Design. (**A**) Stimuli for the eight experimental conditions formed a repeated-measures 2 (emotion) × 2 (source) × 2 (modality) factorial design; Note: for privacy and copyright concerns, face and donut images here were taken by the lab instead of actual images from the image sets. For the same reasons, scene images used are not shown. (**B**) Examples olfactory (left) and visual (right) trials with an odor/sweat and a face/scene image presented before a food image.

Analogous to animal paradigms of STFP, we presented social cues immediately before the presentation of food objects in a 2-alternative-forced-choice (2AFC) task of food healthiness judgment (Fig. 1). Besides manipulating emotion (disgust or neutral), we also included a factor of source (human/social signal or non-human/primary elicitor). By contrasting responses to social (human) signals of disgust (faces and sweat) against responses to primary elicitors of disgust (synthetic odors and natural scenes), we would exclude general disgust effects, thereby isolating specific effects of social disgust. Furthermore, by including a modality factor (olfactory vs. visual), we would pit olfactory responses against visual responses to accentuate modality-selectivity (i.e., olfactory primacy) in social signaling of food choices, akin to the phenomenon of STFP in animals that is dependent on olfactory cues. Therefore, in a 2-by-2-by-2 (emotion-by-source-by-modality) factorial experimental design combined with functional magnetic resonance imaging (fMRI), we tested the hypothesis that social signals (especially chemosignals) would improve food healthiness judgement by recruiting key social and emotional areas in the brain.

## Methods

### Participants

Eighteen healthy female participants took part in the study, who had with normal olfaction and normal or corrected-to-normal vision, no history of neuropsychological problems, and no current use of psychotropic medications. Normal olfactory function was determined based on participants’ self-reported sense of smell and objective assessment (e.g., odor intensity and pleasantness ratings) during a lab visit. Individuals showing aberrant olfactory performance or with nasal infections/allergies were excluded from participating in the study. Only females were recruited due to their presumed sensitivity to chemosignals in the sweat^24,41^. Menstrual cycles (indexed by days since the onset of the last menses) of the participants were fairly evenly distributed across the month [Mean (SD) = 16.3 (8.7) days]. No dietary changes were required other than refraining from consuming anything with a strong smell or flavor within 30 minutes of the experiment. All participants provided informed consent to take part in the study, which was approved by the University of Wisconsin-Madison Institutional Review Board. The experiment was performed in accordance with the approved guidelines and regulations. Two subjects who failed to perform the task were excluded, leaving 16 subjects (mean age, 21; range, 18–29 years) in the final sample.

### Stimuli

Stimuli consisted of neutral and disgust stimuli that were either social (human) signals or primary (non-human) elicitors in either olfactory or visual modality, forming a 2 (emotion) × 2 (source) × 2 (modality) factorial design (Fig. 1A). As discussed below, participants rated the stimuli on a visual analog scale (VAS) of disgust from 0 (not at all) to 10 (extremely disgusting).

#### Visual stimuli

Primary visual elicitors (animals) included six images of animals, three neutral (depicting birds and fish) and three disgust-provoking (depicting cock roaches and bugs). Images were selected from the International Affective Picture Set (IAPS^42^) and the Object Categories Set^43^ (for copyright concerns, images are not shown in Fig. 1A). All images were grey-scaled, equated for size, luminance, and contrast across the sets using the SHINE toolbox^44^. Disgust ratings for disgust images [Mean (SD) = 6.70 (1.73)] and neutral images [1.94 (1.80)] were consistent with the emotion manipulation and differed significantly (*p* < 0.001).

Social visual stimuli (faces) included six face pictures, three expressing neutral and three disgust emotion. Images were selected from the Karolinska Directed Emotional Faces (KDEF), grey-scaled with frontal views in a consistent background^45^. For privacy and copyright concerns, faces shown in Fig. 1A were taken by the lab (not actual faces from the KDEF). Disgust ratings for disgust faces [Mean (SD) = 7.69 (2.20)] and neutral faces [1.36 (1.46)] were consistent with the emotion manipulation and differed significantly (*p* < 0.001).

#### Olfactory stimuli

Primary olfactory elicitors (odors) included synthetic odorous chemicals: three neutral odorants [acetophenone (5% l/l), rose oxide/RO (5%) and α-pinene (10%)] and three disgust odorants [trimethylaminuria/ (5%; rotten fish), valeric acid (7.5%; sweaty socks), and butyric acid (7.5%; rotten eggs)]. Concentrations for the above odorants were determined based on systematic piloting in the lab to achieve comparable, moderate intensity. Disgust ratings for disgust odors [Mean (SD) = 7.28 (1.07)] and neutral odors [2.57 (1.96)] were consistent with the emotion manipulation and differed significantly (*p* < 0.001).

Social olfactory stimuli (sweat).  Axillary sweat was collected form 14 Caucasian male donors. We chose male donors to provide sweat given their larger apocrine glands compared to woman^46^. The donors all provided informed consent to take part in the study, which was approved by the University of Wisconsin-Madison Institutional Review Board. All methods were carried out in accordance with relevant guidelines and regulations. All donors were healthy heterosexual nonsmokers (mean age, 19.8; range, 18–29 years), who had undergone strict dietary (no odorous food intake, no alcohol or smoke, etc.) and behavioral restrictions (no use of deodorants and scented products, no sexual activity, no strenuous exercise, etc.) for 2 days before and on the day of the sweat donation session to minimize extraneous odors in their sweat^23,24^. Emotions were induced by having the donors watch disgust video clips (21 minutes of disgusting scenes, e.g., reality show “Fear Factor” scenes of people eating worms, vomit, and disgusting food) or neutral clips (for 27 minutes of scenes of nature, e.g., landscape and animals) while their sweat was collected. The disgust and neutral sessions were separated by a week in a counterbalanced order across donors. During sweat collection, donors wore a new T-shirt (provided by the experimenter) and a 10 × 10 cm sterile absorbent compress pad (Cutisorb, BSN medical GmbH & Co KG, Hamburg, Germany) under each armpit. Sweat pads were cut into 8 parts and frozen at −22 °C in a freezer (for no more than 10 months before being presented in the experiment). The sweat pads weighed significantly heavier after watching the video [before: Mean (SD): = 4.95 (0.24) g; after: 5.26 (0.55) g; *t* (13) = 2.98, *p* = 0.005], but did not weigh differently between the two emotion conditions before (*p* = 0.16) or after (*p* = 0.31) watching the video. Disgust ratings (0–10) for disgust sweat [Mean (SD) = 2.44 (2.18)] and neutral sweat [1.48 (1.67)] indicated that both were perceived equally neutral (*p* = 0.14), akin to the nature of sweat odors and in keeping with previous reports^24,26^.

#### Food images

Images of food objects (healthy and unhealthy) were taken from Object Categories Set^43^ as targets in the food judgment task. Healthy food images consisted of 8 apple, 8 juice, and 8 multigrain bread images; and unhealthy food images consisted of 8 donut, 8 cake, and 8 cookie images. An independent sample (*N* = 12) made food healthiness ratings on these food images on a VAS (0: extremely unhealthy; 100: extremely healthy). The ratings for the two sets of food pictures confirmed their assigned categories. Scores for the healthy food set [Mean (SD) = 61.60 (8.63)] and the unhealthy food set [Mean (SD) = 8.63 (7.03)] were significantly different between each other and from the neutral midpoint (i.e., 50), *t*’s > 3.24, *p*’s < 0.01. Each image was repeated once in the experiment. Image assignment was fully counterbalanced across participants.

### Procedures

#### Experimental paradigm

Subjects underwent a food judgment task in the scanner. At the beginning of each trial, a grey fixation crosshair was displayed for 2000 ms. In an olfactory trial, a cue reading “Sniff Now” then appeared for 300 ms, followed by a 2-second sweat/odor delivery with a blank frame displayed on the screen (Fig. 1B); In a visual trial, a “Watch Now” cue appeared after the crosshair, followed by a face/animal image for 2 seconds (Fig. 1C). Upon stimulus offset (in both trials), a food picture was presented for 700 ms, to which subjects made a two-alternative-forced choice (“healthy” or “unhealthy”) with a button box. To note, a ninth condition that delivered air only was included to serve as an experimental control condition. Each condition contained 12 trials, which recurred with a fixed stimulus onset asynchrony of 14.1 s. Stimulus order was pseudo-randomized such that no condition was repeated over three trials in a row.

Visual stimuli were presented through a goggles system (Avotec, Inc., FL) linked to the presentation computer, with visual clarity calibrated for each participant. Images were displayed centrally with a visual area of 4.3° × 6.0°. Odor stimuli and odorless air were delivered at room temperature using an MRI-compatible sixteen-channel computer-controlled olfactometer (airflow set at 1.5 L/min). When no odor was being presented, a control air flow was on at the same flow rate and temperature. This design permits rapid odor delivery in the absence of tactile, thermal, or auditory confounds^47–49^). Stimulus presentation and response recording were executed using COGENT software (Wellcome Dept., London, UK) as implemented in MATLAB (Mathworks, Natick, MA).

#### Respiration measurement

During scanning, respiration data were acquired in all subjects using a BioPac MP150 system and accompanying AcqKnowledge software (BioPac Systems, CA) with a breathing belt affixed to the subject’s chest to record abdominal or thoracic contraction and expansion. Offline data analysis was conducted in Matlab, after low-pass filtering (0.5 Hz) to eliminate MRI scanning artifacts. Specifically, sniff waveforms were baseline-adjusted by subtracting the mean activity in the 1000 ms preceding sniff onset, and then averaged across each condition. Sniff inspiratory volume, peak amplitude, and latency to peak were computed for each condition in Matlab.

### Behavioral statistical analysis

We applied signal detection theory analysis on the 2AFC performance and extracted *d’* (Z_hit_ − Z_false alarm_) to indicate discrimination between healthy and unhealthy food and *β* (${e}^{({{\rm{Z}}{hit}}^{2}{-}{{Zfalse\; alarm}}^{2})/2}$) to indicate biases in judgment (*β* > 1 would indicate a bias to judge food items as unhealthy) (Stanislaw & Todorov, 1999). Reaction times (RTs) were also extracted and trimmed by excluding responses over two SDs above the individual mean RT or less than 100 ms^50,51^. Repeated measures analyses of variance (ANOVAs; with Greenhouse–Geisser corrections) with the three experimental factors—emotion (disgust/neutral), source (social/primary), and modality (visual/olfactory)—were performed on *d’*, *β*, and RT. A repeated ANOVA of emotion and source was also performed on respiration parameters to rule out possible confounds related to variations in sniffs across conditions.

### Imaging acquisition and analysis

Gradient-echo T2-weighted echoplanar images (EPI) were acquired with blood-oxygen-level-dependent (BOLD) contrast on a 3T GE MR750 MRI scanner, using an eight-channel head coil with sagittal acquisition. Imaging parameters were TR/TE = 2350/20 ms; flip angle = 60°; field of view, 22 mm; slice thickness 2 mm; slice spacing 1 mm; in-plane resolution/voxel size, 1.72 × 1.72 mm; and matrix size, 128 × 128. A total of 655 volumes were obtained over the experimental run. A high resolution T1-weighted anatomical scan was acquired at a resolution of 1 × 1 × 1 mm^3^. Finally, a field map was acquired with a gradient echo sequence, which was coregistered with EPI images to correct EPI distortions due to susceptibility.

Six “dummy” volumes from the beginning of the session were discarded in order to allow stabilization of longitudinal magnetization. Imaging data were preprocessed using SPM12 (http://www.fil.ion.ucl.ac.uk/spm/software/spm12/) as implemented in Matlab. Images were slice-time corrected and spatially realigned to the first volume of the session, followed by field map correction. Output EPIs were spatially normalized to a standard EPI template. Normalized EPI images were resliced to 2 × 2 × 2 mm^3^ voxels and smoothed with a 6-mm full-width half maximum Gaussian kernel. Normalization was based on Diffeomorphic Anatomical Registration Through Exponentiated Lie algebra (DARTEL^52^).

Next, imaging data were analyzed in SPM12 using the general linear model (GLM). Nine vectors of onset times were created, corresponding to the eight experimental conditions and the air condition. These vectors were coded as delta functions and convolved with a canonical hemodynamic response function (HRF) to form nine event-related regressors of interest. Condition-specific temporal and dispersion derivatives of the HRF were also included to allow for such variations in the HRF. Six movement-related vectors (derived from spatial realignment) were included as regressors of no interest to account for motion-related variance. The data were high-pass filtered (cut-off, 128 s), and an autoregressive model (AR1) was applied. Model estimation yielded condition-specific regression coefficients (*β* values) in a voxel-wise fashion for each subject. In a second step (a random-effects analysis), subject-specific contrasts of these β values were entered into one-sample t tests, resulting in group-level statistical parametric maps of the T statistic (SPM).

#### Regions of interest (ROIs)

We applied ROI analyses on the second (group) level following the first-level whole brain analyses. Based on the extant literature, we focused on a set of a priori ROIs implicated in social and emotion processing, including limbic/prelimbic areas (amygdala, insula, and OFC) and the FFA. Importantly, to isolate modality-selective substrates for olfactory versus visual social signals, we also examined sensory perceptual regions—visual (the inferior occipital and temporal cortices) and olfactory cortices (anterior and posterior piriform cortices /APC and PPC; the olfactory OFC/OFColf). Effects were corrected for multiple comparisons across small volumes of interest (SVC; *p* < 0.05 FWE) based on anatomical ROI masks. As for brain-behavioral associations, to guard against unrealistically high correlations (“voodoo” correlations; Vul *et al*., 2009) forced by statistically corrected thresholds, we also considered effect in the ROIs that reached a heuristic threshold (*p* < 0.001, 10 voxel extent; Lindquist and Mejia, 2015; Eklund *et al*., 2016). Anatomical masks for amygdala and the primary olfactory cortices (APC/PPC) were manually drawn in MRIcro^53^, on the group mean structural T1 image, with reference to a human brain atlas^54^. Due to their less demarcated borders, the other regions were defined by major meta-analysis maps: the OFColf (a higher-order olfactory cortex) and the anterior insula were defined by an olfactory neuroimaging meta-analysis (8-mm spheres around the peak voxels; OFC: −24, 30, −10/28, 34, −12; anterior insula: −30, 18, 6/36, 24, −2^55^), the FFA and occipital face area (OFA) defined by the Neurosynth (www.neurosynth.org) meta-analysis map of faces, and the OFC (non-specific to the OFColf) and insula by the Neurosynth meta-analysis map of disgust. All coordinates reported correspond to Montreal Neurological Institute space.

## Results

### Behavioral data

**Figure 2:**
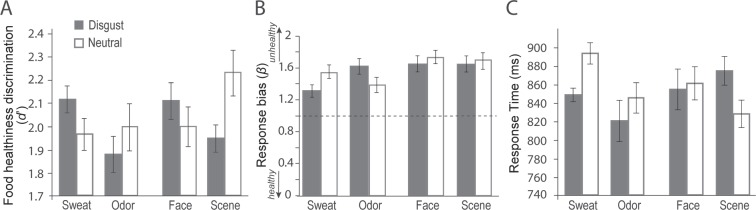
Behavioral results. (**A**) Food healthiness discrimination (*d*’) indicated opposite effects of social and nonsocial disgust (vs. neutral) stimuli: improvement by social disgust in contrast to impairment by nonsocial disgust. (**B**) Response bias measure (*β*) indicated less bias to “unhealthy” responses following disgust sweat and more bias to “unhealthy” responses following disgust odor. The dotted line indicates no response bias (*β* = 1). (**C**) Food judgment RTs indicated speeded responses by olfactory disgust (vs. neutral) stimuli. Error bars =+/−S.E.E. (individually adjusted SEM).

A three-way ANOVA (emotion × source × modality) on food healthiness discrimination (*d’*) yielded a significant interaction between source and emotion [*F* (1, 15) = 8.56, *p* = 0.01; Fig. 2A], but no other significant effects. Breaking the interaction down by source, we observed that primary disgust (vs. neutral) stimuli, in both visual and olfactory modalities, decreased *d’* and thus impeded discrimination between healthy and unhealthy food [*t* (15) = −3.16, *p* < 0.01; disgust *d’* = 1.91 (0.78); neutral *d’* = 2.11 (0.62)] whereas an opposite, marginally significant trend emerged for the social stimuli: social disgust (vs. neutral) stimuli increased *d’* [*t* (15) = 1.89, *p* = 0.08; disgust *d’* = 2.11 (0.72); neutral *d’* = 1.98 (0.77)] and thus improved food healthiness discrimination.

As illustrated in Fig. 2B, potentially owing to the context of disgusting smells and pictures, there was a general response bias to judge the food items as unhealthy across all eight conditions (*p*’s < 0.01). A similar three-way ANOVA on *β* showed a three-way interaction [*F* (1,15) = 4.52, *p* = 0.05; Fig. 2B], in addition to a marginal main effect of source [*F* (1,15) = 3.91, *p* = 0.06]. Follow-up ANOVAs (emotion × source) revealed no effects in the visual modality (*p*’s > 0.50) but a strong interaction effect in the olfactory modality [*F* (1, 15) = 8.79, *p* = 0.01]. Follow-up *t*-tests for olfactory stimuli further revealed reduced *β* in the disgust (vs. neutral) sweat condition, *t* (15) = −2.22, *p* < 0.05, suggesting that disgust sweat dampened the bias in judging food as unhealthy. As illustrated in Fig. 2B, disgust (vs. neutral) odor appeared to increase *β*, but the effect failed to reach statistical significance, *t* (15) = 1.72, *p* = 0.11.

Finally, a similar three-way ANOVA was performed on RTs, which yielded a significant interaction between modality and emotion [*F* (1,12) = 14.00, *p* < 0.005; Fig. 2C], in addition to a marginal main effect of modality [*F* (1, 15) = 4.04, *p* = 0.07]. Follow-up tests in the olfactory modality indicated that relative to neutral stimuli, olfactory disgust (vs. neutral) stimuli (across social and primary conditions) speeded RTs [*t* (15) = −4.58, *p* < 0.001; disgust RT = 834 (225) ms; neutral RT = 869 (231) ms]. In contrast, as illustrated in Fig. 2C, there appeared to be some slowdown of RT following visual disgust (vs. neutral) stimuli [disgust RT = 864 (214) ms; neutral RT = 844 (211) ms], which nonetheless failed to reach significance [*t* (15) = 1.45, *p* = 0.16].

### Respiration data

Two-way ANOVAs (emotion × source) on the sniff parameters (inspiratory volume, peak amplitude, and latency to peak) indicated no simple or interaction effects of emotion and source, *p*’s > 0.1. These results thus ruled out possible sniff-related confounds.

### Neuroimaging data

#### Neural processing of general social olfactory signals

**Figure 3:**
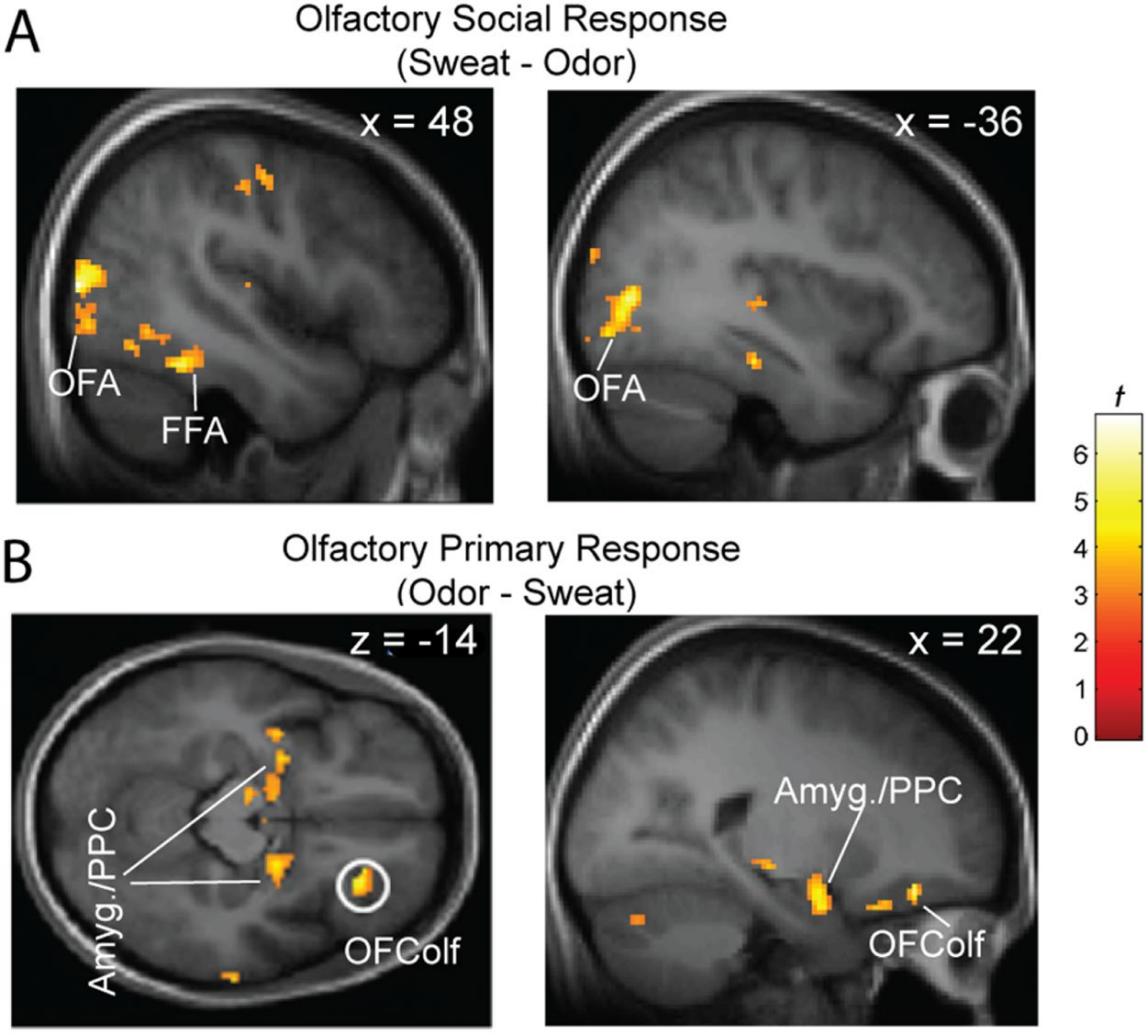
Neural substrates for social and primary olfactory processing. (**A**) General social (vs. primary) olfactory stimuli activated face-processing areas (FFA and OFA). (**B**) General primary (vs. social) olfactory stimuli activated odor-processing areas (OFColf, PPC, and amygdala). Group statistical parametric maps (SPMs) are superimposed on the group mean T1 image (display threshold *p* < 0.005 uncorrected). Amyg. = amygdala.

First, we examined neural areas associated with general social (vs. primary) olfactory processing by contrasting sweat versus odor trials [Sweat (Disgust + Neutral) − Odor (Disgust + Neutral)]. We identified greater response to sweat than odor stimuli in the right FFA (44, −46, −20; *Z* = 3.71, *p* = 0.006 SVC) and the left occipital face area (OFA; −36, −84, −8; Z = 3.37, *p* = 0.006 SVC; Fig. 3A). In comparison, the opposite contrast [Odor (Disgust + Neutral) − Sweat (Disgust + Neutral)] isolated prototypical olfactory areas, including the PPC (−28, 0, −16; *Z* = 3.43, *p* = 0.02 SVC), olfactory OFC/OFColf (26, 34, −14; *Z* = 3.96, *p* = 0.005 SVC), and amygdala (18, −4, −20; *Z* = 3.86, *p* = 0.008 SVC; Fig. 3B). These reliable olfactory effects validated our experiment design and stimulus presentation.

#### Neural processing of olfactory social disgust signals

**Figure 4:**
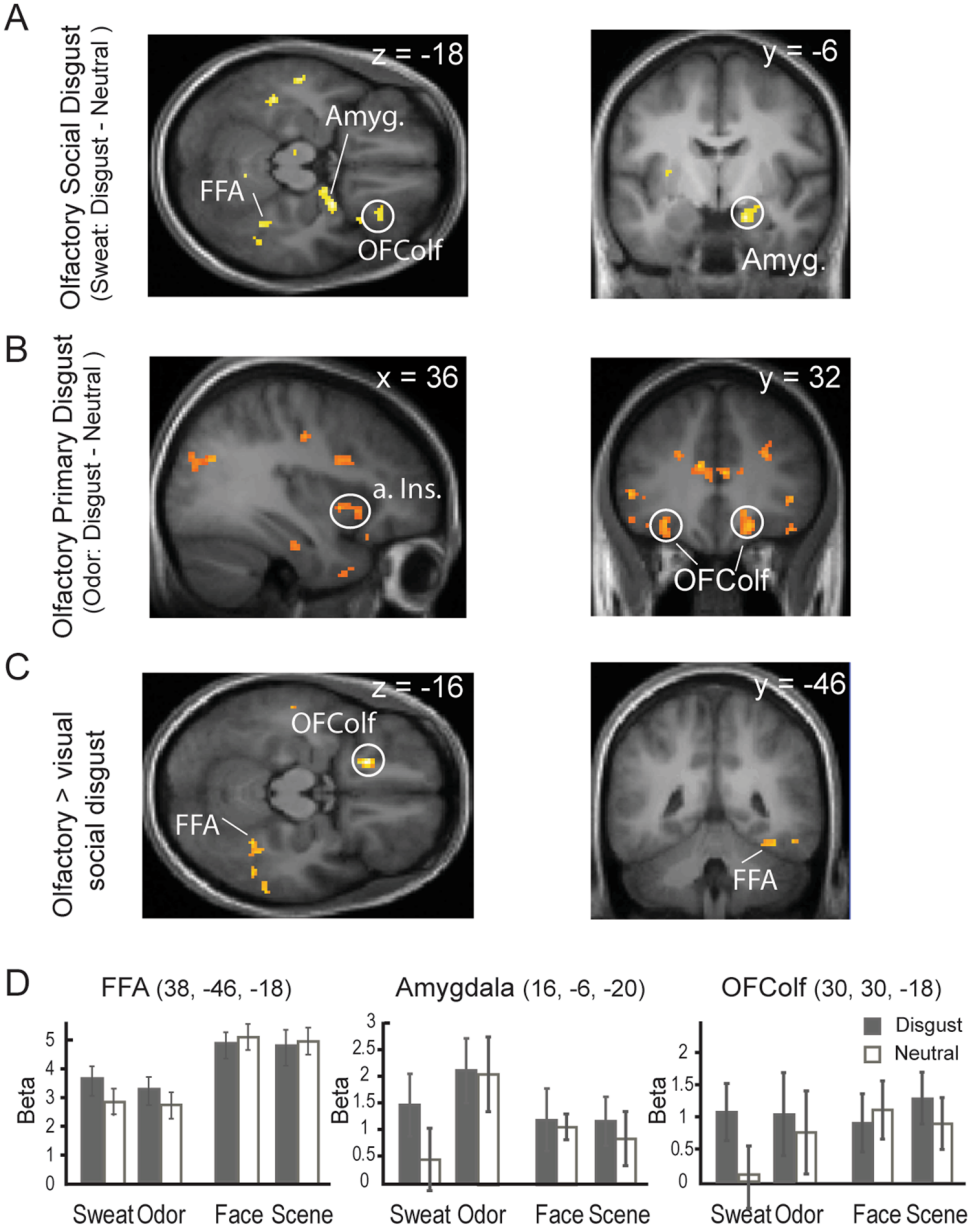
Neural substrates for social and primary disgust processing. (**A**) Olfactory social disgust (vs. neutral) processing involved the right OFColf and amygdala, in addition to the right FFA cluster identified above in the general contrast. (**B**) Olfactory primary disgust (vs. neutral) processing engaged the right anterior insula and bilateral OFColf. (**C**) Preferential processing of olfactory (vs. visual) social disgust was localized to the right FFA and bilateral OFColf (only the left cluster shown). (**D**) Bar graphs of beta estimates in response to the eight conditions for the FFA, amygdala, and OFColf. Group SPMs are superimposed on the group mean T1 image (display threshold p < 0.005 uncorrected). Amyg. = amygdala; a. Ins. = anterior insula. Error bars =+/−S.E.M.

Next, we isolated specific substrates of social chemosignaling of disgust by contrasting disgust and neutral sweat (Disgust Sweat - Neutral Sweat). Importantly, we applied an exclusive mask of disgust versus neutral odor (*p* < 0.05 uncorrected) to the contrast to rule out general olfactory disgust processing. Similar to the contrast above, significant responses were observed again in the right FFA (38, −46, −18; *Z* = 3.15, *p* = 0.027 SVC; Fig. 4A). In addition, effects also emerged in the right amygdala (16, −6, −20; *Z* = 3.53, *p* = 0.02 SVC) and marginally, in the right OFColf (30, 30, −18, *Z* = 3.04, *p* = 0.08 SVC), suggesting that these regions were involved in specific processing of chemosignals of disgust as opposed to general sweat cues.

In comparison, substrates related to specific processing of primary disgust odor (Disgust Odor – Neutral Odor) were localized in the anterior insula (30, 22, 2; *Z* = 3.42; *p* = 0.03 SVC) and marginally, in the left OFColf (−30, 32, −14; *Z* = 3.21, *p* = 0.057 SVC; Fig. 4B), consistent with the extant literature of negative olfactory processing^55^. Note, a similar exclusive mask (Disgust Sweat – Neutral Sweat, *p* < 0.05 uncorrected) was applied to this contrast to remove general olfactory disgust processing.

#### Preferential processing of olfactory versus visual social disgust

Furthermore, we isolated areas preferentially responsive to olfactory (vs. visual) social disgust signals using a contrast of [Sweat (Disgust − Neutral) − Face (Disgust − Neutral)]. Greater response to chemosignals than facial signals of disgust emerged in the right FFA (38, −46, −18, Z = 3.00, *p* = 0.037 SVC) and marginally, the bilateral OFColf (−20, 24, −16/32, 30, −14, Z = 4.15/2.93, *p* = 0.075/0.095 SVC; Fig. 4C). However, the opposite contrast [Face (Disgust − Neutral) − Sweat (Disgust − Neutral)] failed to isolate any significant effects in the ROIs, and even with a very lenient threshold (*p* < 0.01 uncorrected), only a small cluster in the anterior insula emerged (−36, 14, 6, Z = 2.34, *p* < 0.01 uncorrected).

#### Brain-behavior association in social chemosignaling of disgust

Finally, to elucidate how neural processing of chemosignals of disgust contributed to food healthiness judgment, we assessed associations between differential neural responses (Disgust Sweat − Neutral Sweat) and the corresponding signal detection indices (differential *d’* and *β*). A simple regression of the contrast (Disgust Sweat − Neutral Sweat) on differential *d*’ isolated a positive correlation between differential right (posterior) FFA and differential *d*’ (38, −62, 0; *r* = 0.80, *Z* = 3.72, *p* < 0.001, k = 11), suggesting that FFA processing of disgust sweat could inform food healthiness discrimination. Another simple regression of the contrast (Disgust Sweat − Neutral Sweat) on differential *β* identified a negative correlation between differential left OFColf response and differential *β* (−24, 22, −10; *r* = −0.77, Z = 3.72, *p* = 0.026 SVC), suggesting that OFColf analysis of disgust sweat could mitigate the bias to judging food as unhealthy.

## Discussion

Performance in the 2AFC food judgment task, including response speed and two key signal detection indices—*d’* (discrimination sensitivity) and *β* (response bias), demonstrated that social disgust signals, especially chemosignals of disgust, facilitated food healthiness decisions. fMRI data further identified the FFA, amygdala, and OFC in supporting chemosignaling of disgust during food judgment. Importantly, the FFA and OFC exhibited preferential response to olfactory versus visual social signals of disgust, converging with the behavioral finding to highlight a primacy of chemosignaling in social communication of food choices, in line with animal findings of STFP.

As indexed by *d*’, primary disgust (vs. neutral) stimuli (regardless of visual or olfactory modality) interfered with food healthiness discrimination, but social (human) disgust (vs. neutral) signals (also in both modalities) yielded an opposite trend of improved discrimination. As indexed by reduced *β*, olfactory (but not visual) social disgust (vs. neutral) signals attenuated a response bias of judging food items as unhealthy. Finally, as indexed by RT data, olfactory (but not visual) disgust (regardless of primary or social) cues speeded up food judgment, relative to neutral cues. Taken together (at the group level), these results suggest that in the presence of olfactory social disgust (vs. neutral) signals, discrimination between healthy and un-healthy food can be faster, more accurate, and less biased. Overall, this rather comprehensive facilitation by olfactory social disgust contrasts with the effect of visual social disgust on *d*’ (discriminability) alone.

It is important to note that *d*’ and *β* indices for the olfactory modality converged to indicate a significant interaction between source and emotion in food judgment, suggesting that olfactory disgust cues from primary and social sources exert qualitatively distinct effects on food decisions. That is, akin to affective priming effects, primary olfactory disgust (i.e., disgusting odors) tends to disrupt food judgment by reducing accuracy and worsening the bias to judge food as unhealthy. By contrast, social olfactory disgust (arising in response to primary disgust stimuli) is likely to improve food judgment accuracy and reduce the negative bias. Additional correlation analyses further revealed a marginal correlation between differential (Disgust Sweat − Neutral Sweat) *d*’ and *β* (*r* = −0.48, *p* = 0.058), suggesting consistent effects of disgust chemosignals on healthiness judgment at the individual level. Nonetheless, differences in these signal detection measures were not correlated with differential RTs (*r* = −0.07/−0.18, *p*’s > 0.62), presumably due to speed-accuracy trade-offs at the individual level. This beneficial effect is consistent with the phenomenon of STFP such that a chemosignal (e.g., the chemical CS_2_ in the breath of a rat) can enhance a receiver animal’s preference of a food that accompanies the chemosignal. Critically, this food preference can occur even when the food is aversive or the sender of the chemosignal is ill^7,56^, highlighting the notion that such chemosignaling involves information transmission as opposed to mere affective priming^56^.

In keeping with this notion, neural data in rodents suggest that chemosignals for STFP, including CS_2_, are processed not only by the accessory olfactory system as pheromones that are charged with affect and biological instinct but also by canonical olfactory pathways as olfactory sensory inputs^8,10^. Here, our study isolated the FFA, amygdala, and OFColf in the chemosignaling of disgust in food judgement. That is, like rodents, both social/emotional areas and olfactory sensory regions are involved in human chemosignaling, accentuating the possibility that chemosignaling involves the communication of both social/emotional and sensory information.

Specifically, our contrasts between sweat- and odor-elicited responses revealed that general sweat processing recruits face-processing areas (the FFA and OFA) in comparison to primary olfactory processing that engages typical olfaction-proficient regions, including the primary and higher-order olfactory cortices—PPC and OFColf—and the amygdala (a secondary olfactory region^57^. Therefore, compared to primary olfactory stimuli, sweat cues are not potent olfactory but rather strong social signals. However, as revealed by direct contrasts between disgust and neutral sweat and as illustrated in Fig. 4D, this notion appears to apply to neutral sweat primarily. Not only is the FFA especially responsive to disgust (vs. neutral) sweat, the amygdala and OFColf also exhibit preferential response to disgust (vs. neutral) sweat. Moreover, the OFColf and, to some extent, the amygdala respond almost equally strongly to disgust sweat and as to odors, being nonetheless minimally responsive to neutral sweat (Fig. 4D). Therefore, disgust sweat can activate olfactory regions, in addition to face regions. Overall, social and nonsocial chemoreceptive disgust stimuli engage convergent and divergent substrates. They diverge in their sensory perceptual substrates, with primary olfactory disgust activates the primary olfactory cortex (PPC) and social olfactory disgust the low- to intermediate-level face areas (OFA/FFA). Nevertheless, they converge in the OFColf, a region critical for object appraisal and valuation^58,59^. These results combined with the brain behavior associations (between FFA and OFColf responses to disgust and signal detection indices—*d*’ and *β*) led us to speculate that olfactory social disgust communicates social information to influence food judgment in the receiver, by recruiting the FFA to activate social cue processing and the OFC to facilitate value-based decision making.

It is striking that sweat, a chemosensory input, would reliably activate the visual cortex (i.e., OFA/FFA). Nonetheless, it echoes previous neuroimaging studies where signals in the sweat (e.g., regarding mating and sickness) similarly activated the FFA^29,40^. Faces communicate pivotal social information such that face processing would be critically implicated in social communication, and face processing areas serve as key nodes of the social network^35,36^. Furthermore, the FFA can participate in social perception in an amodal manner. For example, the FFA is recruited in recognizing a person’s identity based on the voice, presumably via visuo-auditory cortico-cortical connections^60^. While direct visuo-olfactory cortico-cortical connections are unknown, visuo-olfactory communication can transpire via sensory relays through the OFC and amygdala^48^, which could mediate the participation of FFA in chemosignaling of disgust. The OFA, a low-order face processing area, is not as prominent as the FFA in the social brain, and so its involvement in chemosignaling may reflect strong feedback from the FFA. Alternatively, we suspect that a strong synergy between face and sweat cues would underpin this strong face-related response: in the context of repeated presentation of faces and sweat, the face processing areas could be especially sensitive to social cues to the extent that a sweat cue alone could engage multiple face areas.

Furthermore, while the FFA exhibited greater response to faces than sweat in general (Fig. 4D), in keeping with its primal function of face perception, differential response in the FFA was observed for olfactory but not visual social disgust (vs. neutral) cues. This phenomenon, combined with the behavioral finding of greater effects of olfactory (vs. visual) social disgust, seems to suggest that chemosignals (vs. facial signals) of disgust contain privileged biological information and thus elicit potent neural response, resulting in a strong behavioral impact. This effect aligns with the notion that nonverbal social cues are communicated via selective sensory channels^14–17^. Given the inherent association between olfaction and feeding, the archaic sense of olfaction may assume a privileged channel for social communication of food choices, highlighting an olfactory primacy in human STFP.

As for limitations of the study, we acknowledge that participants’ basic olfactory function was not assessed using a standardized odor test such that subtle olfactory impairment could not be fully excluded. Nevertheless, as the participants were neither patients nor older adults, the likelihood of deviations from normosmia would be low. In addition, information of participants’ sexual orientation was not attained. To the extent that the study did not concern the mating aspect of social chemosignaling, the possibility that sexual orientation could modulate the effect of chemosignaling on food choices was not assessed.

In sum, we demonstrate that like rodents and other animals, humans may also use chemosignals (of disgust) to inform food choices in other individuals. Another person’s chemical messages may outperform visual signals in helping us to choose healthy food. This powerful chemosignaling of disgust engages a complex neural network that integrates regions underpinning social, emotional, and olfactory processing, suggesting that a multi-facet operation is at play during human olfactory social communication. Future research using connectivity analysis is warranted to further define the organization of and connections within this network. Interestingly, the confluence of food, sweat, and social company seems to epitomize a cherished, time-honored tradition in all human societies—eating together with family and friends. Perhaps it is the chemosignals transmitted around the dinner table that are to be credited for the wellbeing of our society, both physically and psychologically.

## Data Availability

Data generated from the current study are available from the corresponding author on reasonable request.

## References

[1] Carels RA, Harper J, Konrad K (2006). Qualitative perceptions and caloric estimations of healthy and unhealthy foods by behavioral weight loss participants. Appetite.

[2] Provencher V, Polivy J, Herman CP (2009). Perceived healthiness of food. If it’s healthy, you can eat more!. Appetite.

[3] Tirindelli R, Dibattista M, Pifferi S, Menini A (2009). From pheromones to behavior. Physiological reviews.

[4] Touhara Kazushige, Vosshall Leslie B. (2009). Sensing Odorants and Pheromones with Chemosensory Receptors. Annual Review of Physiology.

[5] Katz LB, Dill LM (1998). The scent of death: chemosensory assessment of predation risk by animals. Ecoscience.

[6] Leinders-Zufall T (2000). Ultrasensitive pheromone detection by mammalian vomeronasal neurons. Nature.

[7] Galef BG, Mason JR, Preti G, Bean NJ (1988). Carbon disulfide: a semiochemical mediating socially-induced diet choice in rats. Physiology & Behavior.

[8] Munger SD (2010). An olfactory subsystem that detects carbon disulfide and mediates food-related social learning. Current biology.

[9] Shepherd, G. M. *Neurogastronomy: how the brain creates flavor and why it matters* (Columbia University Press, 2011).

[10] Maier JX, Blankenship ML, Barry NC, Richards SE, Katz DB (2014). Stability and flexibility of the message carried by semiochemical stimuli, as revealed by devaluation of carbon disulfide followed by social transmission of food preference. Behavioral neuroscience.

[11] Bushdid C, Magnasco MO, Vosshall LB, Keller A (2014). Humans can discriminate more than 1 trillion olfactory stimuli. Science.

[12] Laska M, Genzel D, Wieser A (2005). The number of functional olfactory receptor genes and the relative size of olfactory brain structures are poor predictors of olfactory discrimination performance with enantiomers. Chemical senses.

[13] Laska M, Seibt A, Weber A (2000). ‘Microsmatic’primates revisited: olfactory sensitivity in the squirrel monkey. Chemical Senses.

[14] Hall JA, Coats EJ, LeBeau LS (2005). Nonverbal behavior and the vertical dimension of social relations: a meta-analysis. Psychological bulletin.

[15] App B, McIntosh DN, Reed CL, Hertenstein MJ (2011). Nonverbal channel use in communication of emotion: How may depend on why. Emotion.

[16] Hertenstein MJ, Holmes R, McCullough M, Keltner D (2009). The communication of emotion via touch. Emotion.

[17] Ekman P, Friesen WV, Osullivan M, Scherer K (1980). Relative Importance of Face, Body, and Speech in Judgments of Personality and Affect. Journal of Personality and Social Psychology.

[18] Semin GR, De Groot JHB (2013). The chemical bases of human sociality. Trends in Cognitive Sciences.

[19] Lundström, J. N. & Olsson, M. J. In *Vitamins & Hormones* Vol. 83, 1–23 (Elsevier, 2010). 10.1016/S0083-6729(10)83001-8 PMC3593650 20831940

[20] Frumin I (2015). A social chemosignaling function for human handshaking. Elife.

[21] Olsson MJ (2014). The scent of disease: human body odor contains an early chemosensory cue of sickness. Psychological science.

[22] Chen D, Haviland-Jones J (2000). Human olfactory communication of emotion. Perceptual and motor skills.

[23] de Groot JHB, Smeets MAM, Kaldewaij A, Duijndam MJA, Semin GR (2012). Chemosignals communicate human emotions. Psychological science.

[24] de Groot JHB (2015). A sniff of happiness. Psychological science.

[25] Li W (2014). Learning to smell danger: acquired associative representation of threat in the olfactory cortex. Frontiers in behavioral neuroscience.

[26] Mujica-Parodi LR (2009). Chemosensory cues to conspecific emotional stress activate amygdala in humans. PLoS One.

[27] Prehn A, Ohrt A, Sojka B, Ferstl R, Pause BM (2006). Chemosensory anxiety signals augment the startle reflex in humans. Neuroscience letters.

[28] Zhou W, Chen D (2009). Fear-related chemosignals modulate recognition of fear in ambiguous facial expressions. Psychological science.

[29] Regenbogen C (2017). Behavioral and neural correlates to multisensory detection of sick humans. Proceedings of the National Academy of Sciences.

[30] Rozin P, Fallon AE (1987). A perspective on disgust. Psychol Rev.

[31] Chapman HA, Anderson AK (2012). Understanding disgust. Ann NY Acad Sci.

[32] Fusar-Poli P (2009). Functional atlas of emotional faces processing: a voxel-based meta-analysis of 105 functional magnetic resonance imaging studies. J Psychiatry Neurosci.

[33] Sabatinelli D (2011). Emotional perception: meta-analyses of face and natural scene processing. Neuroimage.

[34] Pessoa L (2008). On the relationship between emotion and cognition. Nat Rev Neurosci.

[35] Adolphs R (2009). The social brain: neural basis of social knowledge. Annu Rev Psychol.

[36] Allison T, Puce A, McCarthy G (2000). Social perception from visual cues: role of the STS region. Trends Cogn Sci.

[37] Lundstrom JN, Boyle JA, Zatorre RJ, Jones-Gotman M (2008). Functional neuronal processing of body odors differs from that of similar common odors. Cereb Cortex.

[38] Prehn-Kristensen A (2009). Induction of empathy by the smell of anxiety. PLoS One.

[39] Pause BM (2012). Processing of Body Odor Signals by the Human Brain. Chemosens Percept.

[40] Zhou W, Chen D (2008). Encoding human sexual chemosensory cues in the orbitofrontal and fusiform cortices. J Neurosci.

[41] de Groot JHB, Semin GR, Smeets MAM (2014). Chemical communication of fear: A case of male–female asymmetry. Journal of experimental psychology: general.

[42] Lang, P. J., Bradley, M. M. & Cuthbert, B. N. International affective picture system (IAPS): Technical manual and affective ratings. *NIMH Center for the Study of Emotion and Attention* 39–58 (1997).

[43] Konkle T, Brady TF, Alvarez GA, Oliva A (2010). Conceptual distinctiveness supports detailed visual long-term memory for real-world objects. J Exp Psychol Gen.

[44] Willenbockel V (2010). Controlling low-level image properties: The SHINE toolbox. Behav Res Methods.

[45] Lundqvist, D., Flykt, A. & Öhman, A. *The Karolinska Directed Emotional Face*s (1998).

[46] Doty RL, Orndorff MM, Leyden J, Kligman A (1978). Communication of gender from human axillary odors: relationship to perceived intensity and hedonicity. Behavioral biology.

[47] Lorig TS, Elmes DG, Zald DH, Pardo JV (1999). A computer-controlled olfactometer for fMRI and electrophysiological studies of olfaction. Behav Res Methods Instrum Comput.

[48] Novak LR, Gitelman DR, Schuyler B, Li W (2015). Olfactory-visual integration facilitates perception of subthreshold negative emotion. Neuropsychologia.

[49] Forscher EC, Li W (2012). Hemispheric asymmetry and visuo-olfactory integration in perceiving subthreshold (micro) fearful expressions. J Neurosci.

[50] Krusemark EA, Li W (2013). From early sensory specialization to later perceptual generalization: dynamic temporal progression in perceiving individual threats. J Neurosci.

[51] Krusemark E. A., Li W. (2011). Do All Threats Work the Same Way? Divergent Effects of Fear and Disgust on Sensory Perception and Attention. Journal of Neuroscience.

[52] Ashburner J (2007). A fast diffeomorphic image registration algorithm. Neuroimage.

[53] Rorden C, Brett M (2000). Stereotaxic display of brain lesions. Behav Neurol.

[54] Mai, J. K., Assheuer, J. & Paxinos, G. *Atlas of the Human Brain* (Thieme, 1997).

[55] Seubert J, Freiherr J, Djordjevic J, Lundstrom JN (2013). Statistical localization of human olfactory cortex. Neuroimage.

[56] Galef BG, Wigmore SW, Kennett DJ (1983). A failure to find socially mediated taste aversion learning in Norway rats (R. norvegicus). J Comp Psychol.

[57] Carmichael ST, Clugnet MC, Price JL (1994). Central olfactory connections in the macaque monkey. Journal of Comparative Neurology.

[58] Berridge KC, Kringelbach ML (2013). Neuroscience of affect: brain mechanisms of pleasure and displeasure. Curr Opin Neurobiol.

[59] LeDoux JE (1995). Emotion: clues from the brain. Annu Rev Psychol.

[60] Kriegstein Katharina von, Kleinschmidt Andreas, Sterzer Philipp, Giraud Anne-Lise (2005). Interaction of Face and Voice Areas during Speaker Recognition. Journal of Cognitive Neuroscience.

